# Diabetes and tuberculosis: a review of the role of optimal glycemic control

**DOI:** 10.1186/2251-6581-11-28

**Published:** 2012-12-20

**Authors:** Asfandyar Khan Niazi, Sanjay Kalra

**Affiliations:** 1 Shifa College of Medicine, H-8/4 Islamabad, Pakistan; 2 Bharti Hospital and B.R.I.D.E, Karnal, India

**Keywords:** Oral hypoglycemic drugs, Anti-tubercular treatment, Insulin, Epidemic, Glycemic control

## Abstract

Developing countries shoulder most of the burden of diabetes and tuberculosis. These diseases often coexist. Suboptimal control of diabetes predisposes the patient to tuberculosis, and is one of the common causes of poor response to anti-tubercular treatment. Tuberculosis also affects diabetes by causing hyperglycemia and causing impaired glucose tolerance. Impaired glucose tolerance is one of the major risk factors for developing diabetes. The drugs used to treat tuberculosis (especially rifampicin and isoniazid) interact with oral anti-diabetic drugs and may lead to suboptimal glycemic control. Similarly some of the newer oral anti-diabetic drugs may interact with anti-tuberculosis drugs and lower their efficacy. Therefore diabetes and tuberculosis interact with each other at multiple levels – each exacerbating the other. Management of patients with concomitant tuberculosis and diabetes differs from that of either disease alone. This article reviews the association between diabetes and tuberculosis and suggests appropriate management for these conditions.

## Introduction

Tuberculosis (TB) is one of the most common infectious diseases worldwide. For several decades, the research community has been working for an effective preventive strategy for TB. It is clear that although the current preventive efforts against the spread of TB have lowered its incidence, the problem is far from over. Therefore the focus of research has now shifted to the previously untargeted risk factors involved in the spread of TB. One such factor is diabetes mellitus (DM). It is well known that DM impairs the immunity of patients and therefore is an independent risk factor for infections such as TB.

Reports on the association between DM and TB date back to 1000 A.D.; when Avicenna noted that ‘phthisis’, (Greek: tuberculosis), often complicated diabetes and that the presence of diabetes resulted in an increased risk of developing TB [1]. Another description is seen in the works of Yugimahamuni, a traditional Indian saint. He described the association of DM and TB by a combination of symptoms called ‘meganoikal’. These symptoms included obesity, glycosuria, thirst, incontinence, respiratory symptoms and unconsciousness [2].

Each disease is thought to exacerbate and worsen the outcome for the other. TB is a specific morbidity often associated with DM and is therefore aptly described as a complication of DM [3]. People with diabetes are more susceptible to infections and suffer from relatively severe illness due to their immuno-compromised status [4], with reactivation of older foci of TB rather than through fresh contact [5], and often exhibit lower lobe involvement more commonly than in non-diabetics. Various studies have shown that 5-30% of patients with TB have DM as well [6].

### Diabetes as a risk factor for tuberculosis

The incidence of DM is increasing worldwide, especially in developing countries where TB is most prevalent [7]. Therefore the convergence of these two epidemics is most likely to occur in the places with the least amount of healthcare resources. Diabetes is an independent risk factor for all lower respiratory tract infections [8]. A review [9] found 9 studies in which diabetes was estimated to increase the risk of TB from 1.5 to 7.8 fold. Even though TB is more strongly associated with other immune deficiency diseases such as HIV, as the number of people with diabetes is much greater than that of patients with other immunocompromised states, it make DM a more significant risk factor for TB at the population level [10]. A meta-analysis demonstrated that diabetes was associated with a relative risk of 3.11 of contracting TB [11]. An American study has found that multi-drug resistant TB is associated with DM with an odds ratio of 2.1 [12].

Even though type 2 DM is more prevalent, type 1 DM carries a stronger risk of contracting TB [13]. Other risk factors for developing TB in people with type 1 DM include a low body weight, young age, and poor glycemic control [13].

It is not clear whether DM can affect the presentation of TB. Clinical studies have shown ambiguous results. However patients with concomitant TB and DM may have a higher rate of fever and hemoptysis and atypical radiological images. Some studies reported a higher, while others reported a lower, frequency of cavities in the lungs of people with diabetes as compared to non-diabetics with TB [14].

Some studies have also reported a negative effect of DM on the treatment efficacy and prognosis of TB [14]. However, results are conflicting and there is no clear evidence that DM affects the efficacy of treatment of TB. DM also alters the pharmacokinetics of several anti-TB drugs. Since the efficacy of most anti-TB drugs depends on their plasma concentration, it may explain the negative effect of DM on the treatment of TB [7]. The altered plasma levels may be due to differences in absorption, distribution, metabolism and/or excretion in diabetics. Lower plasma levels of anti-TB drugs are associated with resistance to these drugs which may complicate the course of treatment of TB in people with diabetes [15].

### Tuberculosis as a risk factor for diabetes

The relationship between DM and TB is bi-directional. Tuberculosis may lead to the development of new diabetes cases [16,17]. Studies have shown a high prevalence of diabetes,as well as impaired glucose tolerance, in patients with tuberculosis [18]. Impaired glucose tolerance is a significant risk factor for developing DM. In most of these cases, the impaired glucose tolerance reverts back to normal after successful treatment for TB, however the increased risk of developing DM persists [19].

Active tuberculosis should be a differential diagnosis in patients with enlarged pancreas. TB is a known cause of pancreatitis [20] and tuberculous pancreatitis might reveal itself only after the development of diabetes. Even though a part of the hyperglycemia associated with TB may be attributed to the severe stress associated with the infection itself, however the major factor in this process is hypofunction of the pancreas [19,20]. The role of drug interactions is discussed later.

On the other hand, it has been shown that testing for DM in previously undiagnosed people before the appropriate treatment for TB may lead to an over-diagnosis of DM [20]. TB can lead to an infection-related hyperglycemia which may mimic DM. The hyperglycemia associated with TB often aggravates the glycemic control of diabetics and thus warrants adjustment in the dose of insulin [19]. The dose adjustment should be repeated after the patient has been successfully treated for TB.

### Etiology of the association

Diabetes is associated with a decrease in cellular immunity. There are fewer T lymphocytes and a decreased neutrophil count in diabetics. A reduced T-helper1 (Th1) cytokine response level, TNF alpha production, and IL-1 beta and IL-6 production is also seen amongst people with concomitant diabetes and TB as compared to non-diabetic individuals [21-23]. Th1 cytokines are vital in the control and inhibition of mycobacterium tuberculosis bacilli. This decrease in T lymphocyte number and function is primarily responsible for the susceptibility of diabetics to TB. Macrophage function is also inhibited in individuals with diabetes, with an impairment of the production of reactive oxygen species, and phagocytic and chemotactic function. Hyperglycemia has a direct depressive effect on the respiratory burst. A combination of these dysfunctional processes contributes to an increased risk of TB in diabetes [21,22].

It is worthwhile to mention that both these diseases may simulate the symptoms of the other. Such symptoms that are common to both include lethargy, fatigue, weight loss, fever and loss of appetite. It is not unheard of for people with diabetes to present to the doctor with complaints of worsening of blood glucose control only to find out later that they have TB.

### Management of diabetes

Management of DM in TB should be aggressive. An optimal glycemic control results in a better patient outcome; therefore vigorous efforts should be made to achieve such control. Insulin therapy should be initiated at the outset, using basal bolus regime or premixed insulin. The American Association of Clinical Endocrinologists recommends the use of modern insulins or insulin analogues, as they are more predictable in action and cause less hypoglycemia. The use of traditional human insulins is discouraged [24].

Insulin requirements are high to begin with but fall after a few weeks once glucotoxicity is corrected and infection is controlled. Insulin requirements may rise again as appetite returns to normal and caloric intake increases. Sick patients should be tested for ketonuria [25]. Rapid acting analogues such as aspart insulin may obviate the need for admission in patients with ketonuria [26] and are useful for critically ill patients as well [27].

An average patient will need 1.0 U/kg/day of insulin initially, divided as 60% bolus and 40% basal insulin. In a few weeks, the requirement will come down to 0.5 U/kg/day, and may be met by two or three equal doses of premixed aspart/lispro [28]. In patients with co-existing peripheral neuropathy due to diabetes, it is mandatory to give the patient pyridoxine if isoniazid is to be used.

The rationale for exogenous insulin therapy in patients with type 2 diabetes and active tuberculosis is given below [29]:

1) Severe tuberculosis infection

2) Loss of tissue and function of pancreas

a) pancreatic endocrine deficiency

b) tuberculous pancreatitis

3) a) Requirement of high calorie, high protein diet

b) Need for anabolic effect

4) Interactions of antituberculosis drugs with oral antidiabetic drugs

5) Associated hepatic disease prevents use of oral antidiabetic drugs

Oral hypoglycemic agents are contraindicated in severe tuberculosis but may be used with caution once the disease has settled.

Rifampicin is a potent hepatic enzyme-inducer. It accelerates the metabolism of several oral hypoglycemic agents, especially sulphonylureas and biguanides, and lowers their plasma levels. Therefore it may cause hyperglycemia in diabetic patients using these drugs. In non-diabetics, it augments the intestinal absorption of glucose and may simulate the symptoms of diabetes [30].

Isoniazid, in contrast to rifampicin, inhibits the metabolism of oral hypoglycaemic agents and may lead to an increase in the plasma levels of these drugs. Its main interaction is with sulphonylureas, the action of which it antagonizes and worsens the glycemic control of diabetics on this medication. It also impairs the release and action of insulin leading to hyperglycemia even in non-diabetics [31]. Therefore the dosage of insulin should be adjusted while adding and removing these drugs from the patients’ prescriptions. Dipetidyl protease inhibitors (the gliptins), a comparatively newer class of hypoglycemic agents, have a theoretical possibility of reducing immunocompetence because of their mechanism of action [32]. This effect could possibly worsen the outcome of patients with TB.

## Conclusions

Diabetes and tuberculosis should be treated aggressively with insulin. In case a person with diabetes and active tuberculosis is poorly controlled on oral hypoglycemic agents, it is necessary to switch to insulin. The choice of insulin regime should be based on efficacy, safety, tolerability and convenience.

South Asia is known as the diabetes capital and the tuberculosis capital of the world. Both diseases are linked with each other, and need to be treated together for optimal outcome. A concerted effort on our part can change our designation to the Diabetes CARE capital and Tuberculosis CARE capital of the world.

## Competing interests

The authors declare that they have no competing interests.

## Authors’ contributions

AKN was involved in searching the literature and helped in drafting the article. SK wrote the first draft of the article and edited the article throughout all stages. Both authors read and approved the final manuscript.
